# Evaluation of a Computer-Aided Clinical Decision Support System for Point-of-Care Use in Low-Resource Primary Care Settings: Acceptability Evaluation Study

**DOI:** 10.2196/47631

**Published:** 2024-06-11

**Authors:** Geletaw Sahle Tegenaw, Demisew Amenu Sori, Girum Ketema Teklemariam, Frank Verbeke, Jan Cornelis, Bart Jansen

**Affiliations:** 1 Department of Electronics and Informatics Vrije Universiteit Brussel Brussel Belgium; 2 Faculty of Computing Jimma Institute of Technology Jimma University Jimma Ethiopia; 3 Department of Obstetrics and Gynecology College of Health Science Jimma University Jimma Ethiopia; 4 Interuniversitair Micro-Electronica Centrum Leuven Belgium

**Keywords:** low-resource setting, clinical decision support system, point-of-care instrument, evaluation, user acceptance, structural equation modeling, partial least squares structural equation modeling, decision-making, decision making, decision support, caregiver, users, acceptance, system quality

## Abstract

**Background:**

A clinical decision support system (CDSS) based on the logic and philosophy of clinical pathways is critical for managing the quality of health care and for standardizing care processes. Using such a system at a point-of-care setting is becoming more frequent these days. However, in a low-resource setting (LRS), such systems are frequently overlooked.

**Objective:**

The purpose of the study was to evaluate the user acceptance of a CDSS in LRSs.

**Methods:**

The CDSS evaluation was carried out at the Jimma Health Center and the Jimma Higher Two Health Center, Jimma, Ethiopia. The evaluation was based on 22 parameters organized into 6 categories: ease of use, system quality, information quality, decision changes, process changes, and user acceptance. A Mann-Whitney U test was used to investigate whether the difference between the 2 health centers was significant (2-tailed, 95% CI; α=.05). Pearson correlation and partial least squares structural equation modeling (PLS-SEM) was used to identify the relationship and factors influencing the overall acceptance of the CDSS in an LRS.

**Results:**

On the basis of 116 antenatal care, pregnant patient care, and postnatal care cases, 73 CDSS evaluation responses were recorded. We found that the 2 health centers did not differ significantly on 16 evaluation parameters. We did, however, detect a statistically significant difference in 6 parameters (*P*<.05). PLS-SEM results showed that the coefficient of determination, R^2^, of perceived user acceptance was 0.703. More precisely, the perceived ease of use (β=.015, *P*=.91) and information quality (β=.149, *P*=.25) had no positive effect on CDSS acceptance but, rather, on the system quality and perceived benefits of the CDSS, with *P*<.05 and β=.321 and β=.486, respectively. Furthermore, the perceived ease of use was influenced by information quality and system quality, with an R^2^ value of 0.479, indicating that the influence of information quality on the ease of use is significant but the influence of system quality on the ease of use is not, with β=.678 (*P*<.05) and β=.021(*P*=.89), respectively. Moreover, the influence of decision changes (β=.374, *P*<.05) and process changes (β=.749, *P*<.05) both was significant on perceived benefits (R^2^=0.983).

**Conclusions:**

This study concludes that users are more likely to accept and use a CDSS at the point of care when it is easy to grasp the perceived benefits and system quality in terms of health care professionals’ needs. We believe that the CDSS acceptance model developed in this study reveals specific factors and variables that constitute a step toward the effective adoption and deployment of a CDSS in LRSs.

## Introduction

The use of health information systems has considerably transformed the health care sector in recent years [1]. Proper and coordinated implementation is beneficial to the enhancement of health care delivery [2,3]. An effective clinical decision support system (CDSS); low-cost, point-of-care diagnostics; effective remote clinics; home-based therapies; and improved communication with patients and across health care facilities are among the benefits [4,5]. Even though the implementation of a CDSS at the point of care has sought to improve treatment quality and resource efficiency, its use in low-resource settings (LRSs) has lagged behind due to a variety of restrictions.

In Ethiopia, the health care system is a 3-tiered system organized into primary, secondary, and tertiary levels of care [6]. Primary health care settings include primary hospitals, health centers, and health posts. Recently, an electronic community health information system and district health information software were implemented in Ethiopian public health centers. These tools are commonly used for routine data management tasks. Frontline workers, however, lacked easy access to decision support systems and other similar point-of-care technologies. Paper-based clinical guidelines (CGs), card sheets, and point-of-care charts were the only available resources, and only limited information is documented on the card sheets [7,8]. Delivering evidence-based services at the point of care by capturing the required clinical data, summarizing and processing them in a consistent manner, and constructing a patient flow sheet to monitor and record the progress of care from the paper-based resources were challenging [7,8]. The Ethiopian national maturity health information assessment survey also revealed that there is a lack of health information infrastructure, a lack of decision support and knowledge management systems, and a lack of parameters and metrics for analyzing the impact of data [9].

Thus, introducing and integrating a CDSS with the existing health information system helps deliver appropriate, consistent, and integrated care. To introduce a CDSS in LRSs, we followed a 3-step approach:

- Step 1: A case study (maternal and childcare health services) needs analysis was conducted in LRSs to assess the available point-of-care evidence of the requirements for a CDSS, such as clinical pathways (CPs) or workflows [7,8].
- Step 2: We conducted a state-of-the-art review to investigate strategies and approaches for designing CDSS instruments for LRSs [10]. The aim was to review existing publications in the LRS context to explore recommended approaches and design considerations for building a CDSS.
- Step 3: A CDSS was developed based on the findings of the needs analysis and a review of the state of the art. The CDSS was designed to reduce delays and support frontline workers. The proposed CP algorithm, in particular, aims to find referrals and locally treatable cases by integrating knowledge-based approaches and historical evidence [11].

**Figure 1:**
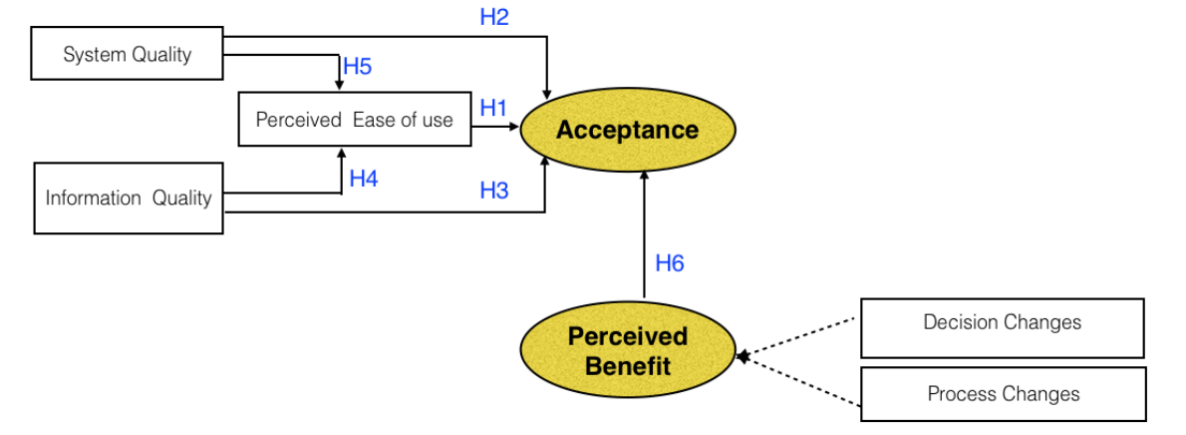
Computer-aided CDSS evaluation model hypotheses. Customized and adopted from Ji et al [20]. CDSS: Clinical decision support system; H: Hypothesis.

The aim of this study was to evaluate the user acceptance of a CDSS in LRSs. Overall, as depicted in Figure 1, this study proposed the following hypotheses to evaluate the user acceptance of the CDSS:

- Hypothesis (H)1: The perceived ease of use has a positive effect on the acceptance of a CDSS in LRSs.
- H2: System quality has a positive effect on the acceptance of a CDSS in LRSs.
- H3: Information quality has a positive effect on the acceptance of a CDSS in LRSs.
- H4: Information quality has a positive effect on the perceived ease of use of a CDSS in LRSs.
- H5: System quality has a positive effect on the perceived ease of use of a CDSS in LRSs.
- H6: Perceived benefits have a positive effect on the acceptance of CDSS in LRSs.

## Methods

### Ethical Considerations

Approval for the research was granted by the Institutional Review Board of the Institute of Health, Jimma University (reference number IHRPGI/467/19).

### Study Settings and Participants

This study was conducted in low-resource primary health care centers, with a specific focus on the maternal and childcare health service units at the Jimma Health Center and the Jimma Higher Two Health Center. Both health centers are situated in Jimma Town, in the Oromia region, Southwestern Ethiopia. Each of them serves up to 40,000 people in its geographical area, accepts referrals from community health posts, and refers patients to the nearest hospital, such as the Shanan Gibe General Hospital and the Jimma University Specialized Hospital. The health centers serve and oversee both inpatient and outpatient cases. The number of personnel in the Jimma Higher Two Health Center is 34 and in the Jimma Health Center is 40, whereas in Ethiopia, the health center’s maternal and child health service unit employs a much smaller number of health professionals, commonly 5-7 nurses and midwives. There were 5 nurses and midwives at the Jimma Health Center and 4 at the Jimma Higher Two Health Center during our investigation. The maternal and childcare health service unit is expected to serve 2000-2500 antenatal care (ANC), pregnant patient care, and postnatal care (PNC) cases annually.

Participants in the CDSS evaluation were health care professionals, such as midwives and nurses, who worked at the maternal and childcare health service unit at the Jimma Health Center and the Jimma Higher Two Health Center. The inclusion and exclusion criteria were as follows:

- Health care professionals were personnel at the maternal and childcare health service unit and were familiar with the existing clinical workflow, as well as volunteering to evaluate the CDSS.
- The ANC, pregnant patient care, and PNC cases that had been pre-recorded on the evaluation day were suitable for retrospective chart review to evaluate the CDSS.
- Both morning and afternoon evaluations were based on the pre-recorded cases from the respective morning and afternoon visits.

The CDSS evaluation was conducted in the health care professionals’ spare time because the number of health care professionals at the maternal and childcare health service unit was limited, and they were so preoccupied and busy with their regular daily activities that it was not feasible to incorporate the evaluation into their routine. The health care professionals completed a questionnaire over the course of a half-day (as a summary of the half-day cases rather than as a case-by-case response), with the morning session taking place from 11:00 to1:00 A.M. and the afternoon session taking place from 5.00 to 18:30 P.M.

The initial evaluation was conducted in August 2022 at the Jimma Health Center. The second round of evaluation took place at the Jimma Health Center and the Jimma Higher Two Health Center from December 20, 2022, until January 15, 2023.

Based on our previous experience [13], obtaining the expected sample size in an LRS was difficult due to a shortage of health care professionals in the maternal and childcare health service unit (usually 4-7).

To determine the optimal strategies, we consulted the existing literature in support of our evaluation study design. Based on the findings of Mburu and Oboko’s study [14], we observed that 79 cases were sufficient to assess the use of mobile health (mHealth) interventions in Kenya. Additionally, Mburu and Oboko [14] also reported that 60 subjects were sufficient to detect the small and medium effects of an exogenous latent variable (independent variable) on an endogenous latent variable (dependent variable), according to the findings of Chin and Newsted [15] and Cohen [16], just as using 40 subjects was sufficient for Goodhue et al [17]. The minimum sample size needed to observe an effect with a given power (ie, the probability of observing a statistically significant result at level *P* if a true effect of a certain magnitude is present) is determined by the effect size. The effect size is associated with the path coefficient between a variable that is assumed to describe a cause and a variable that is assumed to be an effect: values<0.02 indicate no effect, values>0.15 indicate a medium effect, and values>0.35 indicate a large effect [17,18]. Moreover, using 70-80 samples was adequate to model functional brain relationship hypotheses in the study by Sideridis et al [19]. However, Sideridis et al [19] also explicitly noted that sample sizes of 50 participants were associated with a root mean square error of approximation of <0.05, suggesting a satisfactory fit.

The study entailed a proof-of-principle CDSS evaluation using a convenience sample of 7 health professionals. Altogether, we reviewed 73 ANC, pregnant patient, and PNC cases.

### Procedure and Measurement Instrument

A tutorial and a demonstration were provided to the health care professionals at the 2 health centers prior to using the CDSS. The health care professionals used and assessed the CDSS before completing a questionnaire. They used a retrospective chart review, specifically a half-day of pre-recorded patient card sheet data, to evaluate the CDSS. On the basis of pre-recorded cases, the goal was to evaluate how well the CDSS performed in identifying referrals and locally treatable cases that were actually made. The health care professionals then filled out questionnaires to provide their assessments and feedback on the CDSS. Each evaluation questionnaire was completed based on a half-day of ANC, pregnant patient, and PNC cases, as well as the health care professional’s observation of the CDSS reaction to the presented cases. Next, the health care professionals answered a series of 5-point Likert scale items (1=strongly disagree, 2=disagree, 3=neutral, 4=agree, 5=strongly agree) about the CDSS [20]. The measurement instrument consisted of 22 parameters adopted from Ji et al’s [12] evaluation framework. The 22 measurement items were classified into 6 factors: system quality, information quality, service quality, perceived ease of use, user acceptability, and perceived benefits. Furthermore, we automated the questionnaire submission, which was accessed via a mobile phone or a laptop. Electronic questionnaire submission was preferred over paper-based alternatives. However, paper-based questionnaire submissions were used in some cases.

### The CDSS at the Point of Care

We designed and developed a CDSS to meet the requirements of LRSs. An intelligent clinical wizard, minimum data and data readiness, adaptable features, and low-cost infrastructure are some of the notable requirements and prerequisites of LRSs based on our previous results [7]. Our CDSS incorporates both existing knowledge-based guidelines and data-driven evidence to provide the most relevant information for frontline workers at the time of care delivery [11]. The CDSS provides CPs (or workflows) for point-of-care services. The CP is a critical component of a CDSS for identifying referral and locally treatable cases, which is delivered in the form of a concordance table for multicriteria decision analysis and output [11].

The CDSS has the following major goals:

- Delivering automated CPs and computer-assisted pruning and selection.
- Going beyond existing paper-based evidence that is noninteractive and challenging to grasp, the computerized CDSS was designed to be interactive for ease of use and optimal usage.
- Combining existing CGs and historical evidence (data-driven evidence) to generate an adaptable clinical workflow.

**Figure 2:**
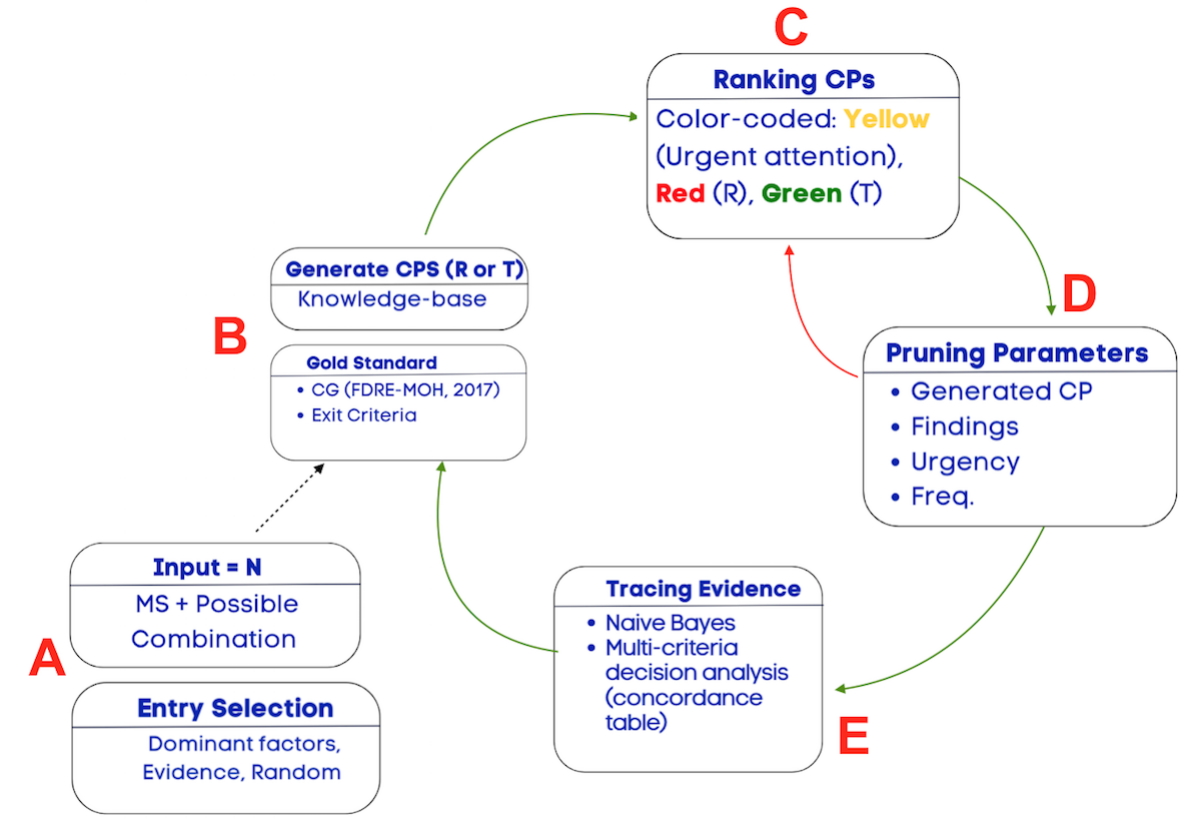
CP-processing workflow. CG: Clinical guideline; CP: Clinical pathway; FDRE-MOH: Federal Democratic Republic of Ethiopia Ministry of Health; Freq.: Frequency; MS: Measured symptom.

To get the most out of services, the CDSS provides an automated, interactively adaptable CP (or workflow). To reduce arbitrariness in entry point selection, the CDSS provides a range of choices for initiating the CP, such as using evidence from historical records, dominant factors, or randomly initiating the signs and symptoms based on CGs. Figure 2A presents additional information about entry point processing.

The process is interactive, and our algorithm uses measured symptoms (MSs) and a combination of MSs to process the CPs:

- First, all CPs based on the first MS are generated, as shown in Figure 2B. CGs are used as the gold standard and criterion for validating the generated CP (also referred to as an exit criterion). If the generated CP is already found on the generated list, the frequency counter is incremented. Otherwise, the generated CP is added (or appended) to the generated list of CPs. Federal Democratic Republic of Ethiopia Ministry of Health version 2017 (FDRE-MOH 2017) is used for CP processing.
- Second, a ranking of CPs is conducted to identify “referral” and “locally treatable” cases. The ranking is color-coded, as shown in Figure 2C, and the ranking criteria are based on CGs.
- Third, the dynamic CP list is pruned, as shown in Figure 2D. CP pruning is based on pruning parameters. If the generated CP list is empty, fall-back and adjustment of the pruning criterion are supported. The pruning process was designed to be interactive, flexible, responsive, and engaging. The user intervention allows for fine-tuning based on domain knowledge and provides trust and understanding for the health care professional. Pruning can also be based on findings if the health care professional requires pruning of specific CP findings. The findings are based on the CGs.
- Fourth, the naive Bayes algorithm and historical records are used to provide data-driven evidence, as shown in Figure 2E. The output is displayed in an easy-to-understand format, using a table to present the evidence. The ranked table provides evidence for assessing various factors, such as symptoms, findings, urgency, CP, CP frequency, accuracy, and prior and posterior probability, to facilitate evidence-based decision-making by the user. Since it provides evidence for analyzing various factors, we refer to it as multicriteria decision analysis. In further detail, the multicriteria output used for decision analysis is displayed in the form of a table, also known as a concordance table. A concordance table is a data (evidence) table used as a cross-reference for integrating evidence from many sources for decision support. In this study, it was used primarily for tracing what evidence was available to support the presented case and identifying the evidence’s source (historical records or knowledge-based evidence). A more detailed step-by-step description of the algorithms is found in Figure 2A-E.
- Finally, the preceding steps are repeated for each additional MS.

**Figure 3:**
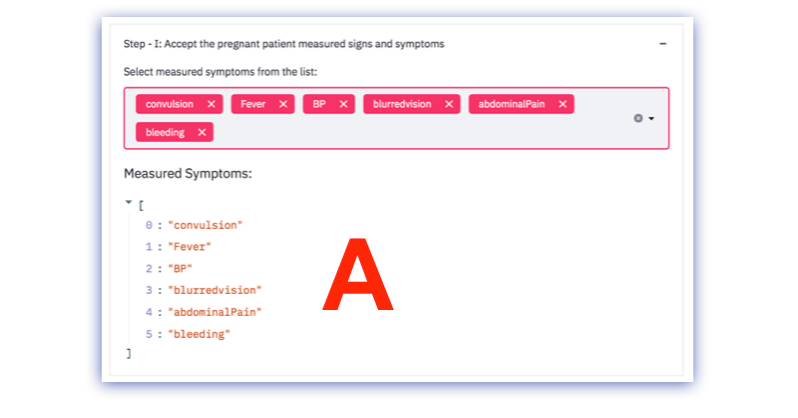
Screenshot of input processing. BP: blood pressure.

**Figure 4:**
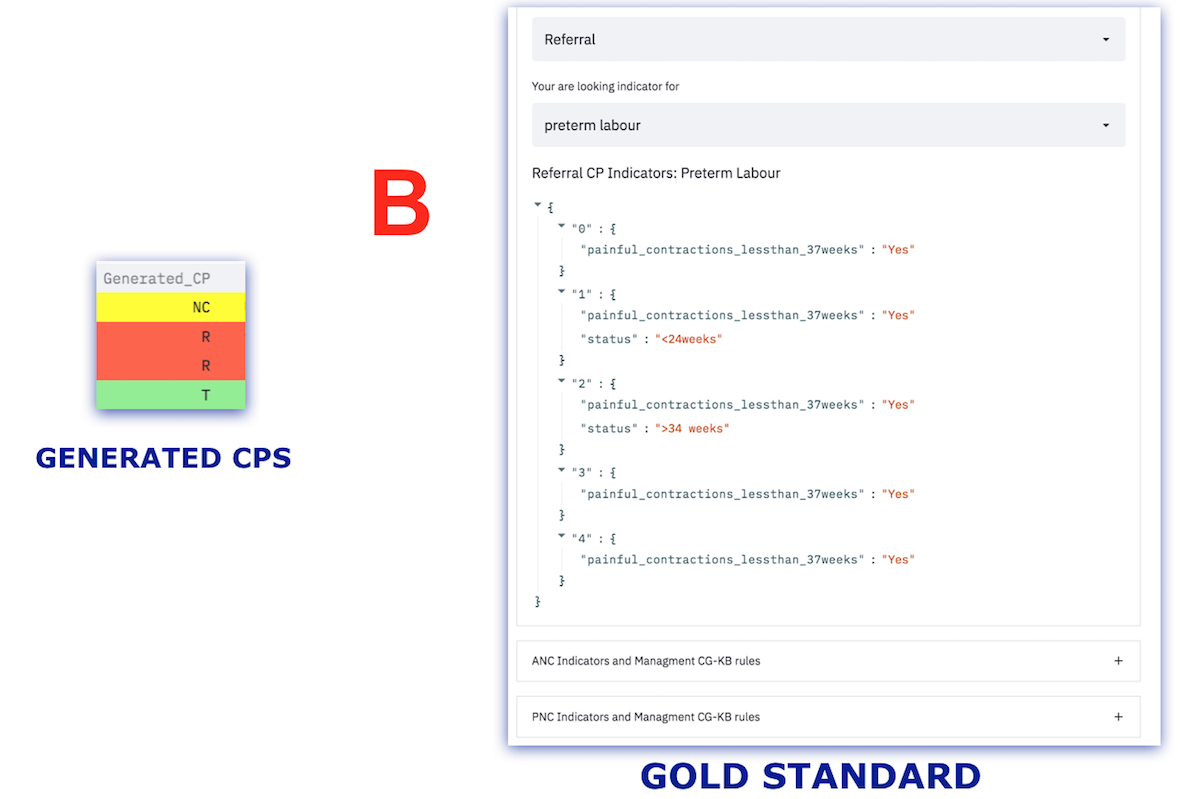
Screenshot of the generated CPs and the gold standard. ANC: antenatal care; CG: clinical guideline; CP: clinical pathway; KB: knowledge base; NC, not classified; PNC: postnatal care; R: referral; T: treatable.

**Figure 5:**
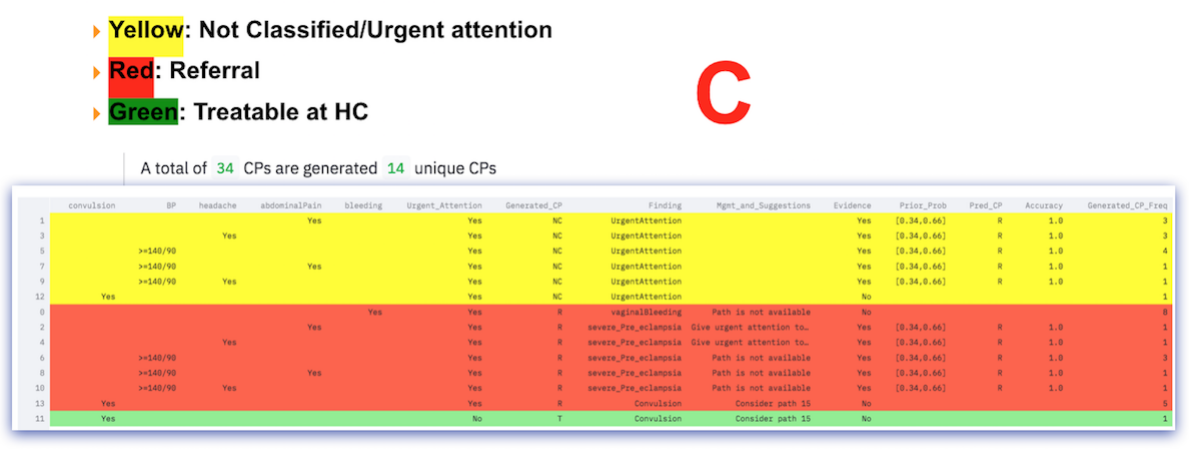
CP ranking. BP: blood pressure; CP: clinical pathway; HC: health center.

**Figure 6:**
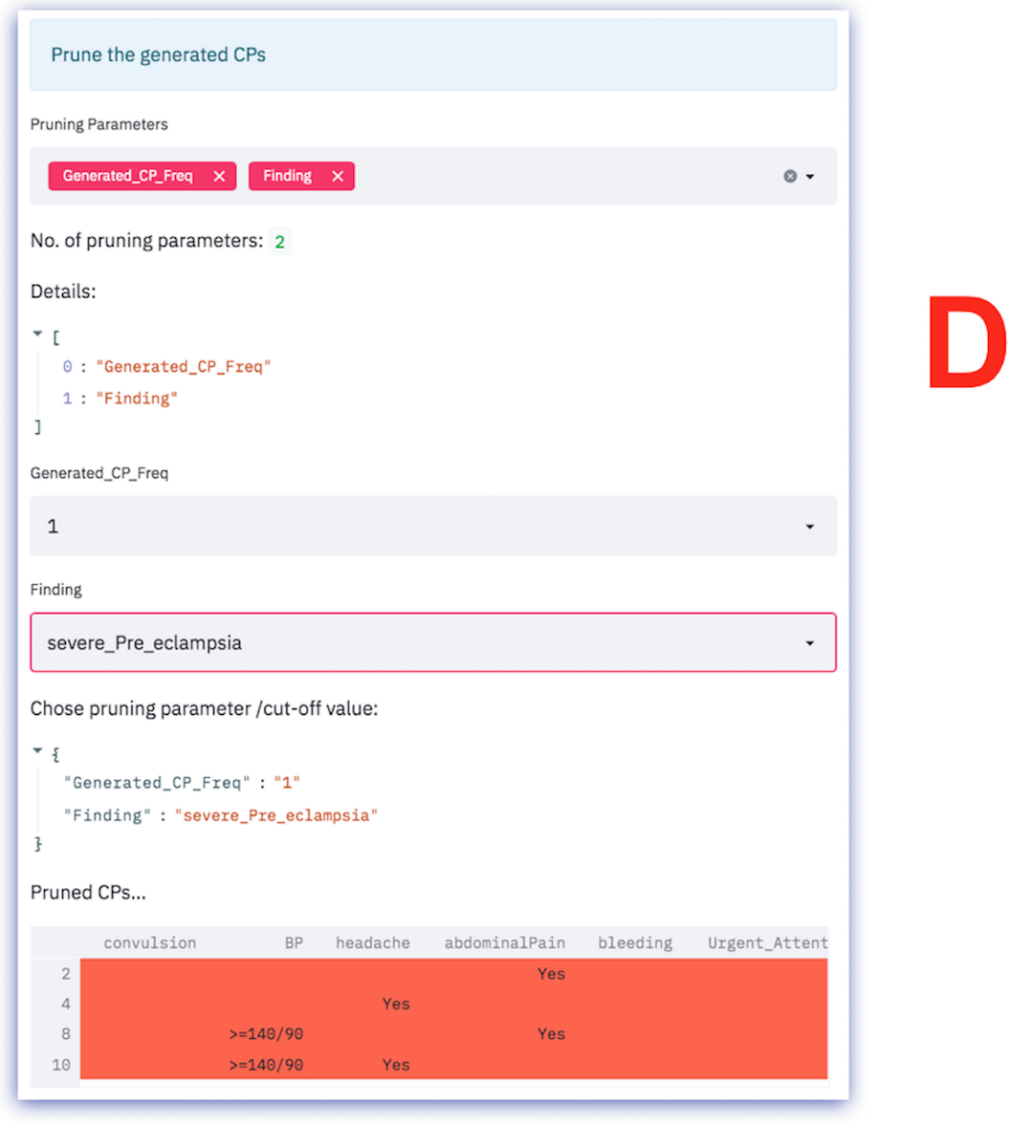
CP pruning. BP: blood pressure; CP: clinical pathway.

**Figure 7:**
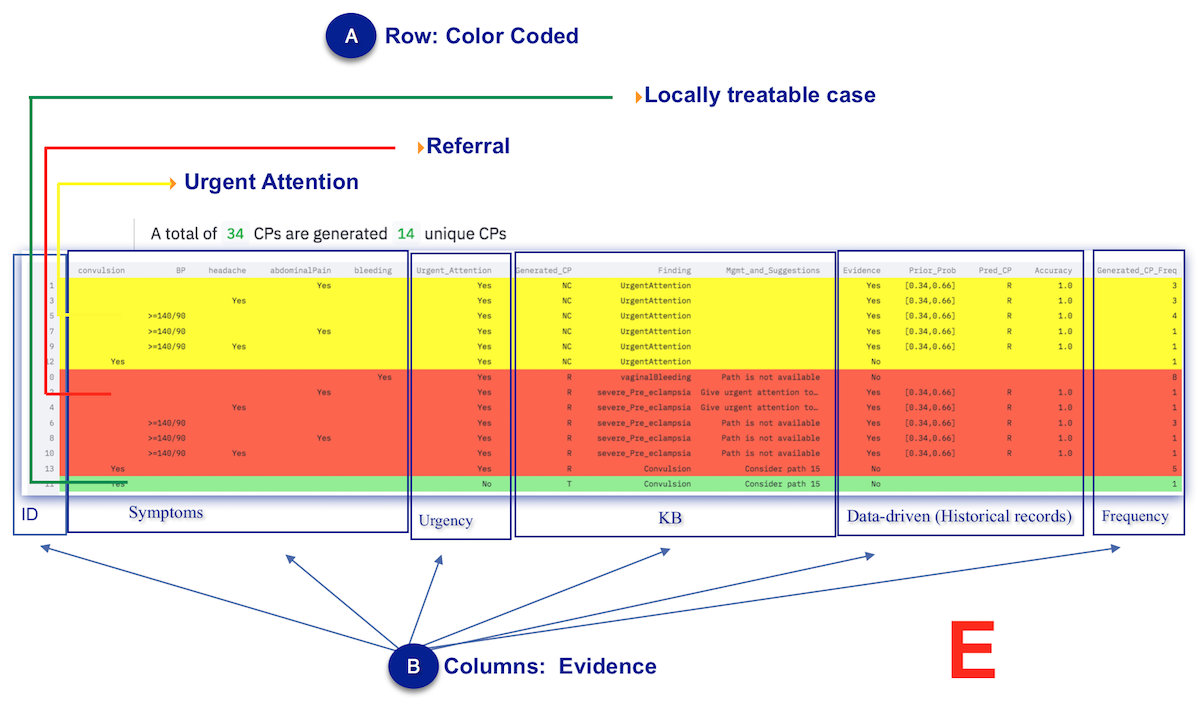
Concordance table. BP: blood pressure; CP: clinical pathway.

In the end, the frontline worker must make the final decision based on the suggestions made by the algorithm. For this study and demonstration, the CDSS focused on 3 use cases, namely pregnant patient care, ANC, and PNC services. The sample user interface screenshot for each step is shown in Figures 3-7.

### Data Analysis

Statistical Package for Social Sciences (SPSS; IBM Corporation) version 26.0 [21], Microsoft Excel [22], Python (version 3.7) [23], and SmartPLS (version 26.0) [24] were used to conduct the analysis and modeling.

We followed the procedures and recommendations of Boone and Boone [25] for the CDSS evaluation based on Likert data analysis. Latent variables were computed by summing the following items:

- The perceived ease of use was a latent variable based on learnability, operability, user interface, data entry, advice to display, and legibility items.
- Response time and stability items were used to assess system quality.
- Information quality was based on security and CP performance items.
- Acceptance included usage, confirmation of expectations, overall quality satisfaction, overall satisfaction, and the intention to use items.
- Perceived benefits were created using decision change (change in order behavior, change in CP) and process change (effectiveness, overall usefulness, adherence to standards, medical quality, and user knowledge and skills) items.

To assess the scale of the CDSS evaluation data set, the validity of the measurement model was checked. Convergent validity was assessed using factor loading and average variance extracted (AVE), with a factor loading threshold of more than 0.70 and an AVE threshold of >0.50 [26,27]. In this study, items with factor loadings of less than 0.70 were candidates for deletion. The internal consistency and reliability of the CDSS evaluation measurement model were assessed using Cronbach α [28] and composite reliability. A recommended value of >.70 for Cronbach α and composite reliability was accepted. We used the heterotrait-monotrait ratio of correlations (HTMT) [26,29] to check the discriminant validity of the measurement model and determined whether the value was less than 0.90 and acceptable. Moreover, perceived benefits are formative second-order construct based on decision changes and process changes. Collinearity was checked to ensure that it did not have a negative impact on the higher-order-construct measurement model, and critical levels of collinearity less than 0.50 were acceptable in this study, as recommended by Hair et al [26].

Following that, item-level and construct-level analyses were performed. On the one hand, an item-level analysis of the CDSS in LRSs between the 2 health centers was conducted. A nonparametric independent-samples statistical test, such as the Mann-Whitney *U* test [30], was used to see whether the 2 health centers were significantly different at the item level. We used the Mann-Whitney *U* test because we could not assume normality in either group and the independent data set observation assumptions were fulfilled, which are preconditions for the use of nonparametric data analysis [31]. Furthermore, there were no significant results from the Shapiro-Wilk test [32] on the normality of our evaluation data set. The significance level used for the inferential statistics was *P*=.05 and a 95% CI level.

On the other hand, we followed the recommendation of Boone and Boone [25] to use Pearson correlation for construct-level (latent variable) correlation analysis. As a result, Pearson correlation [25,33] was used to examine the factors influencing the acceptance of the CDSS in LRSs and the interrelationships between construct factors. In particular, the relationship between system quality and perceived ease of use, information quality and perceived ease of use, user acceptance and perceived benefits, and user acceptance and information and system quality were explicitly explored.

Finally, structural equation modeling (SEM) is a multivariate statistical analysis technique that is used to analyze structural relationships. It is described in the literature as combined factor analysis and regression analysis for discovering relationships between measured variables and latent constructs [34]. There is a debate on how effective it is to discover causation beyond correlation. In papers dealing with applications of the technique, it is commonly used to express a causal hypothesis in a context where there is semantic information available that supports the validity of the hypothesis or at least does not contradict it [12,14,35]. Our study was a pilot study, not a full, cross-sectional analysis, and it intended to promote the use of partial least squares structural equation modeling (PLS-SEM) [26]. PLS-SEM was used to model the acceptance of the CDSS in LRSs, particularly to model the relationship between the CDSS evaluation measured items and construct variables, as well as between multiple construct variables. We noticed that penalized likelihood estimation algorithms based on regularized structural equation modeling (RegSEM) [36,37] and PLS-SEM [26] were the best candidates for our modeling. We preferred PLS-SEM for the following reasons:

- The SmartPLS [26,38] partial least squares (PLS) algorithm was used to analyze the model’s path weight, and it performed well in Mburu and Oboko’s [14] study.
- The variation-based structural equation models do not impose a sample size [39] or normality of distribution constraints [26,38].

Overall, to construct the PLS-SEM model for the CDSS in LRSs, first, composite factor analysis was used to examine the validity of the measurement model, including reliability and validity analysis. The relationships in path models with latent variables were then evaluated using PLS-SEM path analysis and coefficients. Finally, the statistical significance of PLS-SEM results, such as path coefficients, outer weights, Cronbach α, and coefficient of determination (R^2^) values, was determined using bootstrapping [26]. The bootstrapping settings were percentile bootstrap, 2-tailed test type, and significance level=.05.

## Results

### Characteristics

The 7 CDSS evaluators were all female (ie, n=4, 57%, from the Jimma Health Center and n=3, 43%, from the Jimma Higher Two Health Center), who worked as health care professionals (eg, midwives and nurses) in the health centers’ maternal and childcare health service units. In total, 73 CDSS evaluation responses were recorded based on 116 ANC, pregnant patient care, and PNC cases (n=4, 5%, during the first evaluation period and n=69, 95%, during the second evaluation period). The response was 73 since the evaluation response was based on a summary of half-day cases rather than a case-by-case response. The average time for evaluating the CDSS and completing the questionnaire was 52.35 minutes, with the smallest and longest durations being 31 and 98 minutes, respectively. The Jimma Health Center accounted for 65.5% (76/116) cases, while the Jimma Higher Two Health Center accounted for 34.5% (40/116) cases. Furthermore, we observed that each health center handled 4-6 (3%-5%) cases per day on average. Overall, the first round of evaluation lasted 2 days and included 18 ANC, pregnant patient care, and PNC cases in the Jimma Health Center, which is above average. In round 2, there were 75 ANC cases, 7 pregnant patient care cases, and 16 PNC cases during our evaluation period. The second round of evaluation took place in both health centers, the Jimma Health Center and the Jimma Higher Two Health Center.

The computer-aided CDSS evaluation’s mean (SD) score ranged from 4.29 (SD 0.485) to 4.52 (SD 0.503). Table 1 provides more extensive details of each item score.

**Table 1:** Mean Likert scale scores and reliability analysis for computer-aided CDSS^a^ evaluation in LRSs^b^.

Construct and items		Value, minimum (maximum)	Score of 73 CDSS evaluation responses, mean (SD)
**Perceived ease of use**			
	Learnability	2 (5)	4.30 (0.545)
	Operability	3 (5)	4.29 (0.485)
	User interface	3 (5)	4.34 (0.533)
	Data entry	3 (5)	4.40 (0.571)
	Advice display	3 (5)	4.37 (0.589)
	Legibility	1 (5)	4.29 (0.905)
**System quality**			
	Response time	3 (5)	4.38 (0.543)
	Stability	2 (5)	4.38 (0.615)
**Information quality**			
	Security	3 (5)	4.32 (0.550)
	CP^c^ performance	3 (5)	4.37 (0.540)
	Decision change		
	Change in order behavior	2 (5)	4.08 (0.640)
	Change in CP	2 (5)	4.23 (.613)
**Process changes**			
	Effectiveness	3 (5)	4.25 (0.494)
	Overall usefulness	3 (5)	4.23 (0.635)
	Adherence to standards	3 (5)	4.33 (0.502)
	Medical quality	3 (5)	4.29 (0.612)
	User knowledge and skills	2 (5)	4.30 (0.570)
**Acceptance**			
	Usage	3 (5)	4.49 (0.580)
	Confirmation of expectations	2 (5)	4.34 (0.628)
	Satisfaction with overall quality	3 (5)	4.40 (0.571)
	Overall satisfaction	—^d^	4.30 (0.570)
	Intention to use	4 (5)	4.52 (0.503)

^a^CDSS: clinical decision support system.

^b^LRS: low-resource setting.

^c^CP: clinical pathway.

^d^Not applicable.

### CDSS Evaluation Measurement Model

The factor loading of 20 (91%) of 22 items was greater than 0.70. The remaining items, legibility and medical quality, were eliminated since their factor loading value was less than 0.70. All the constructs had Cronbach α values greater than .70, except information quality, for which Cronbach α was .699, which is close to .70. Table 2 provides more information about the measurement model’s construct reliability and validity.

To establish discriminant validity, the HTMT on construct factors was used, and the results showed that all constructs passed the test. Table 3 displays the results of the discriminant validity assessment.

**Table 2:** CDSS^a^ measurement model’s construct reliability and validity.

Construct and items			Convergent validity						Internal consistency and reliability			
			Factor loading (>0.70)		AVE^b^ (>0.50)			Composite reliability (>0.70)			Cronbach α (>.70)	
**Perceived ease of use**						0.588			0.847			.825
	Learnability	0.721		—^c^			—			—		
	Operability	0.738		—			—			—		
	User interface	0.746		—			—			—		
	Data entry	0.856		—			—			—		
	Advise to display	0.836		—			—			—		
**System quality**						0.879			0.869			.863
	Response time	0.934		—			—			—		
	Stability	0.944		—			—			—		
**Information quality**						0.763			0.767			.699
	Security	0.825		—			—			—		
	CP^d^ performance	0.930		—			—			—		
**Decision changes**						0.776			0.712			.712
	Change in order behavior	0.856		—			—			—		
	Change in CP	0.856		—			—			—		
**Process changes**						0.650			0.824			.819
	Effectiveness	0.773		—			—			—		
	Overall usefulness	0.813		—			—			—		
	Adherence to standards	0.896		—			—			—		
	User knowledge and skills	0.762		—			—			—		
**Acceptance**						0.654			0.871			.867
	Usage	0.738		—			—			—		
	Confirmation of expectations	0.819		—			—			—		
	Satisfaction with overall quality	0.806		—			—			—		
	Overall satisfaction	0.846		—			—			—		
	Intension to use	0.815		—			—			—		
**Perceived benefits**												
	Constructed based on decision and process changes	—		0.511			0.848			0.839		

^a^CDSS: clinical decision support system.

^b^AVE: average variance extracted.

^c^Not applicable.

^d^CP: clinical pathway.

**Table 3:** CDSS^a^ discriminant validity assessment.

Constructs	Perceived ease of use	Information quality	Perceived benefits	Perceived user acceptance	System quality
Perceived ease of use	—^b^	—	—	—	—
Information quality	0.855	—	—	—	—
Perceived benefits	0.643	0.852	—	—	—
Perceived user acceptance	0.616	0.877	0.877	—	—
System quality	0.545	0.839	0.618	0.779	—

^a^CDSS: clinical decision support system.

^b^Not applicable.

### CDSS Evaluation Between the 2 Health Centers

The results of the nonparametric Mann-Whitney *U* test based on the 5-point Likert item evaluation data set collected from the Jimma Health Center and the Jimma Higher Two Health Center revealed that the 2 health centers did not differ significantly in the CDSS item-level evaluation factors, except stability (*U*=470.5, *P*=.022), overall usefulness (*U*=451.0, *P*=.012), adherence to standards (*U*=483, *P*=.024), confirmation of expectations (*U*=488.5, *P*=.04), satisfaction with overall quality (*U*=400.5, *P*=.001), and overall satisfaction (*U*=474.5, *P*=.023). The findings of the CDSS evaluation using the Mann-Whitney *U* test are shown in Table 4.

**Table 4:** Mann-Whitney U test results (*P*<.05).

Construct and items		Mean rank		Test statistics^a^	
		Jimma Health Center (n=42)	Jimma Higher Two Health Center (n=31)	Mann-Whitney *U*	Asymptotic significance (2-tailed) *P* value
**Perceived ease of use**					
	Learnability	36.92	37.11	647.5	.962
	Operability	38.75	34.63	577.5	.309
	User interface	37.48	36.35	631.0	.794
	Data entry	37.85	35.85	615.5	.653
	Advice display	36.14	38.16	615.0	.650
	Legibility	34.76	40.03	557.0	.249
**System quality**					
	Response time	33.48	41.77	503.0	.057
	Stability	32.70	42.82	470.5	.022
**Information quality**					
	Security	35.49	39.05	587.5	.409
	CP^b^ performance	35.65	38.82	594.5	.466
**Decision changes**					
	Change in order behavior	36.48	37.71	629.0	.767
	Change in CP	35.51	39.02	588.5	.416
**Process changes**					
	Effectiveness	35.82	38.60	601.5	.489
	Overall usefulness	32.24	43.45	451.0	.012
	Adherence to standards	33.00	42.42	483.0	.024
	Medical quality	34.43	40.48	543.0	.174
	User knowledge and skills	34.50	40.39	546.0	.164
**Acceptance**					
	Usage	32.79	42.71	474.0	.024
	Confirmation of expectations	33.13	42.24	488.5	.04
	Satisfaction with overall quality	31.04	45.08	400.5	.001
	Overall satisfaction	32.80	42.69	474.5	.023
	Intension to use	35.38	39.19	583.0	.381

^a^Grouping variable: health center.

^b^CP: clinical pathway.

### CDSS Evaluation Agreement Score Observation in the Jimma Health Center

Although the total number of observations in the first and second rounds of the CDSS evaluation were not equal, we found a positive mean agreement score increment in the majority of evaluation parameters at the Jimma Health Center, which was calculated using “agree” and “strongly agree” responses. Adherence to the standards agreement score, however, declined from 1.00 to 0.974. The first and second round CDSS evaluation agreement score observations at the Jimma Health Center are shown in Table 5.

**Table 5:** First and second round CDSS^a^ evaluation agreement score observations at the Jimma Health Center.

Construct and items			Round 1: agreement score based on n=4 observations, mean (SD)					Round 2: agreement score based on n=38 observations, mean (SD)		
			Item level		Construct level		Item level			Construct level
**Perceived ease of use**			—^b^		0.708		—			0.982
	Learnability	0.750		—		1.000			—	
	Operability	0.750		—		1.000			—	
	User interface	0.750		—		1.000			—	
	Data entry	0.750		—		1.000			—	
	Advice to display	0.750		—		1.000			—	
	Legibility	0.500		—		0.890			—	
**System quality**			—		0.625		—			0.987
	Response time	0.750		—		1.000			—	
	Stability	0.500		—		0.974			—	
**Information quality**			—		0.750		—			0.974
	Security	0.750		—		0.947			—	
	CP^c^ performance	0.750		—		1.000			—	
**Decision changes**			—		0.750		—			0.960
	Change in order behavior	0.500		—		0.947			—	
	Change in CP	1.000		—		0.974			—	
**Process changes**			—		0.800		—			0.953
	Effectiveness	0.750		—		1.000			—	
	Overall usefulness	0.750		—		0.842			—	
	Adherence to standards	1.000		—		0.974			—	
	Medical quality	0.750		—		0.947			—	
	User knowledge and skills	0.750		—		0.974			—	
**User acceptance**			—		0.750		—			0.958
	Usage	0.750		—		0.947			—	
	Confirmation of expectations	0.500		—		0.974			—	
	Satisfaction with overall quality	0.750		—		0.947			—	
	Overall satisfaction	0.750		—		0.921			—	
	Intention to use	1.000		—		1.000			—	

^a^CDSS: clinical decision support system.

^b^Not applicable.

^c^CP: clinical pathway.

### CDSS Evaluation: Construct Factor Interrelationships

**Figure 8:**
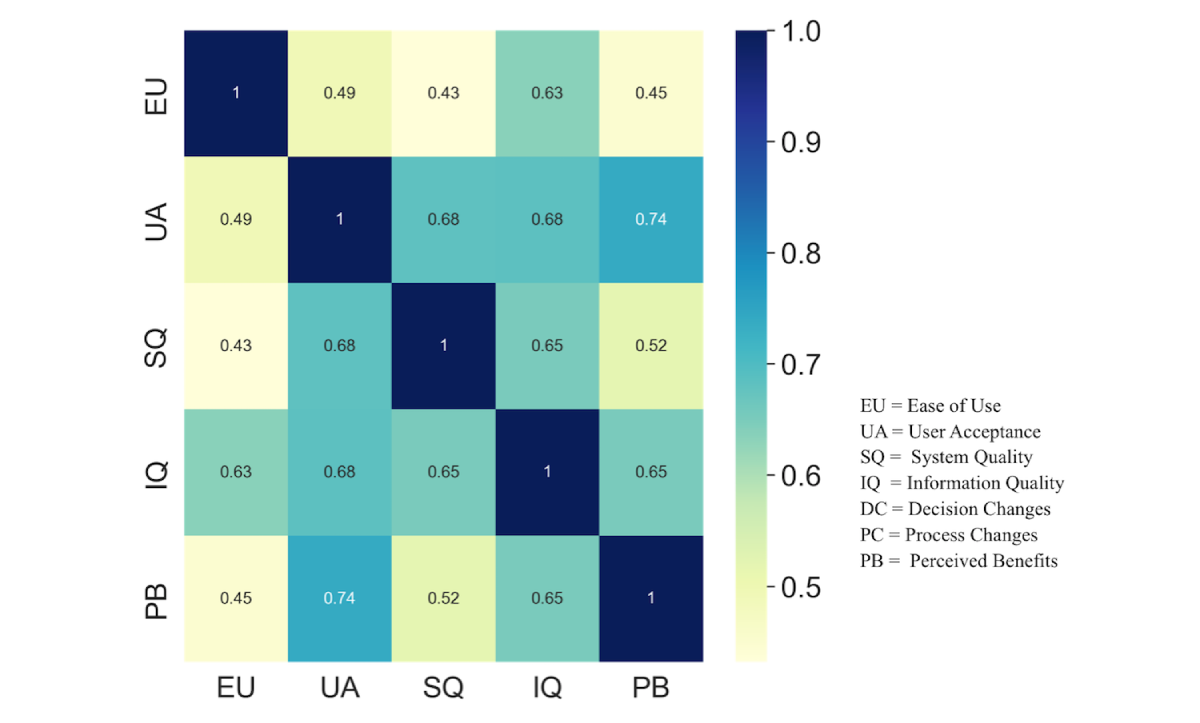
Pearson correlation (N=73). Correlation values ranging from 0.50 to 0.70 are considered moderate, from 0.70 to 0.90 are considered strong, and from 0.9 to 1.0 are considered very strong [33].

We found a significant correlation (r =0.74) between user acceptance and perceived benefits, with perceived benefits as construct factors based on process changes and decision changes. The coefficient of correlation between perceived ease of use and information quality was r=0.63. User acceptance was also correlated with information quality and system quality, with r=0.68. Figure 8 depicts a more detailed Pearson correlation test result.

### Modeling the Acceptance of the CDSS in LRSs

**Figure 9:**
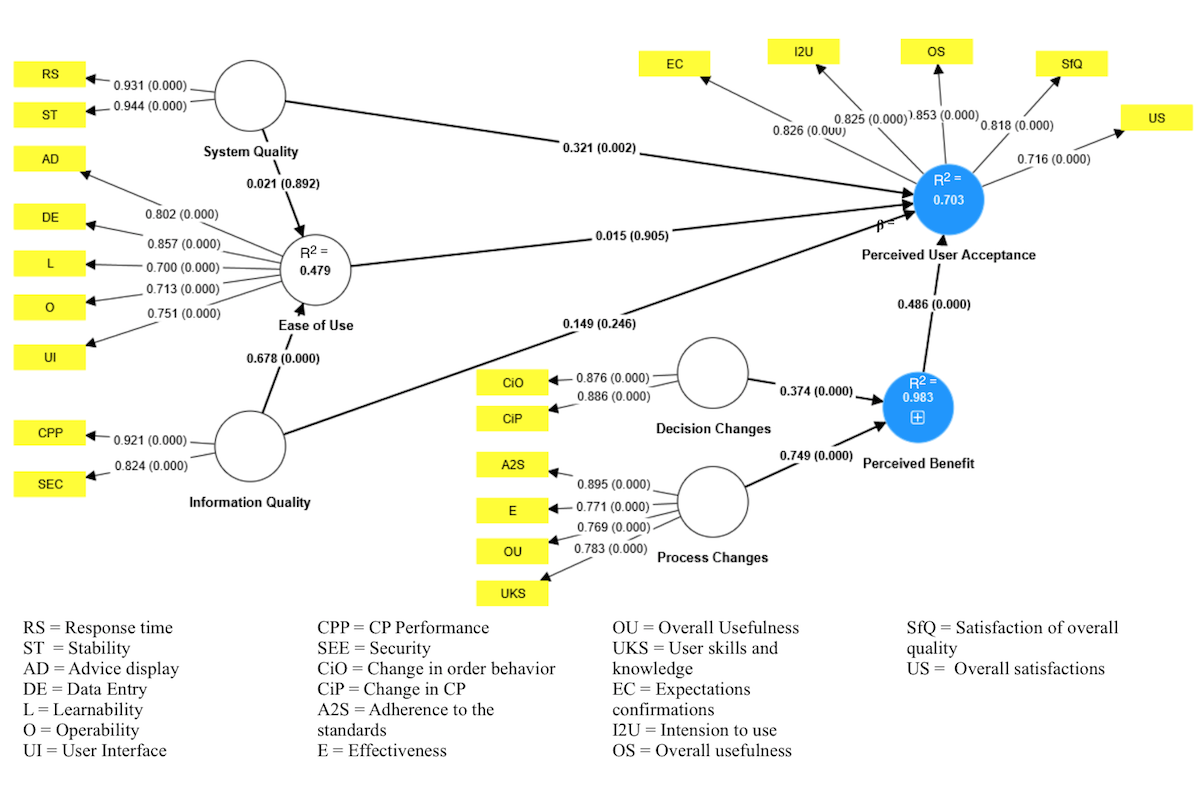
Computer-aided CDSS evaluation PLS-SEM model generated from the computer-aided CDSS Jimma Health Center and the Jimma Higher Two Health Center evaluation data sets, showing path weights (β), *P* values, and coefficient of determination (R^2^). The yellow boxes represent indicators (or parameters). The construct variables are represented by the circle. The path indicates the path weight and *P* value. For example, a 0.321 (.002) value from system quality -> perceived user acceptance shows that β=.321 and *P*=.002. CDSS: clinical decision support system; PLS-SEM: partial least squares structural equation modeling.

The perceived user acceptance coefficient of determination (R^2^) was 0.703, showing that user acceptance is influenced by system quality, information quality, perceived ease of use, and perceived benefits (Figure 9). More precisely, system quality (β=.321, *P*<.05) and perceived benefits (β=.486, *P*<.05) were shown to have a significant influence. However, the perceived ease of use had no positive effect on CDSS acceptance (β=.015, *P*=.91). Information quality also had no positive effect on CDSS acceptance in this study (β=.149, and *P*=.25).

Furthermore, we found that the perceived ease of use was influenced by system quality, and information quality, with an R^2^ value of 0.479. The path coefficient of information quality on the perceived ease of use was β=.021(*P*=.89), and hence, no significant effect was found. The path coefficient of system quality on the perceived ease of use was β=.678 (*P*<.05), that is, a significant influence, whereas the perceived benefits impacted by decision and process changes had an R^2^ value of 0.983. The path coefficients of decision changes and process changes were β=.374 and β=.749, respectively, and were significant (*P*<.05). Figure 9 depicts the path weights, *P* values, and coefficient of determination (R^2^) for the CDSS evaluation PLS-SEM model developed using the CDSS Jimma Health Center and Jimma Higher Two Health Center evaluation data sets. The results, shown in Figure 9, can be interpreted as perceived ease of use -> perceived user acceptance (β=.015 and *P*=.91), for example. Overall, we found that the perceived ease of use had no positive effect on CDSS acceptance (β=.015, *P*=.91) but, rather, on the system quality (β=.321, *P*<.05) and perceived benefits (β=.486, *P*<.05) of the CDSS. Further information is presented in Table 6.

**Table 6:** Hypotheses conclusion based on PLS-SEM^a^ findings (β=.015, *P*=.91).

Hypothesis	Path and relationships	PLS-SEM findings^b^				Conclusion
		β (SD)	*t* Statistics	*P* value		
Hypothesis (H)1: The perceived ease of use has a positive effect on the acceptance of a CDSS^c^ in LRSs^d^.	Perceived ease of use -> acceptance	.015 (0.123)	0.119	.91	Rejected	
H2: System quality has a positive effect on the acceptance of a CDSS in LRSs.	System quality -> acceptance	.321(.102)	3.139	.002	Accepted	
H3: Information quality has a positive effect on the acceptance of a CDSS in LRSs.	Information quality -> acceptance	.149 (0.128)	1.162	.25	Rejected	
H4: Information quality has a positive effect on the perceived ease of use of a CDSS in LRSs.	Information quality -> perceived ease of use	.678 (0.122)	5.558	<.001	Accepted	
H5: System quality has a positive effect on the perceived ease of use of a CDSS in LRSs.	System quality -> perceived ease of use	.021 (0.153)	.135	.89	Rejected	
H6: Perceived benefits have a positive effect on the acceptance of a CDSS in LRSs.	Perceived benefits -> acceptance	.486 (0.115)	4.234	<.001	Accepted	

^a^PLS-SEM: partial least squares structural equation modeling.

^b^Relationships were significant at *P*<.05.

^a^CDSS: clinical decision support system.

^d^LRS: low-resource setting.

## Discussion

### Principal Findings

This study aimed to evaluate a CDSS for use at the point of care in primary care LRSs. The health care professionals in this study evaluated user acceptance of the CDSS.

The Cronbach α scale of 22 items appeared to be internally consistent, exceeding the minimum value of .70 required for acceptable reliability [26-28,32]. In this study, the 2 health centers did not differ significantly in terms of the CDSS’s perceived ease of use, information quality, and perceived benefits (decision changes and process changes). However, we found a significant difference in system quality, such as stability, and perceived user acceptance, such as overall usefulness, adherence to standards, confirmation of expectations, satisfaction with overall quality, and overall satisfaction. This variation could be attributed to the first round of evaluation, which was based on the Jimma Health Center, or to the fact that more cases were observed in the Jimma Health Center than in the Jimma Higher Two Health Center, but more research and analysis are required. Furthermore, based on the first and second rounds of the CDSS evaluation, we observed a positive agreement score increment at the Jimma Health Center. However, this study was unable to observe a change in the Jimma High Two Health Center, since the first round of evaluation was limited to the Jimma Health Center.

This study highlighted a correlation between construct variables using Pearson correlation. The CDSS’s system quality, information quality, and perceived benefits were vital for its acceptance in the LRS. The perceived benefits were based on decision and process changes. In accordance with our results, previous studies have demonstrated that the acceptance and use of mHealth apps in LRSs are influenced by users’ perceptions that are aligned with health needs and expectations [14]. However, in this study, the perceived ease of use was moderately correlated with CDSS acceptance, whereas Ji et al [12] suggested that the perceived ease of use has a significant and direct impact on the acceptance of a CDSS. Figure 8 depicts further information about the Pearson correlation between the construct variables of the CDSS. Overall, we observed a low positive Pearson correlation between the perceived ease of use and acceptance, as well as between system quality and the perceived ease of use, when we considered the strength of correlation classification as in Mukaka [33]. System quality and acceptance, information quality and acceptance, and information quality and perceived ease of use all showed a moderately positive correlation. There was a high positive correlation between perceived benefits and acceptance, supporting Pande et al’s [40] finding that perceived usefulness is significantly correlated to the intention to use.

This study also used PLS-SEM to evaluate several factors that impact the acceptance of a CDSS in LRSs. The result demonstrated that user acceptance is impacted by system quality, information quality, and perceived benefits, with an R^2^ value of 0.703, as shown in Figure 9. The perceived benefits influenced by decision and process changes had an R^2^ value of 0.983, whereas the R^2^ score for the perceived ease of use as impacted by system and information quality was only 0.479. All retained R^2^ values were greater than 0.10, as suggested by Falk and Miller [41]. The R^2^ values of the perceived user acceptance and perceived benefits were substantial, as also indicated by the CDSS results of Cohen [18], who reported R^2^>0.26, and Chin [42], who reported R^2^>0.67. However, according to the criteria of Hair et al [43], R^2^ of perceived benefits is greater than 0.75 and substantial, while R^2^ of the perceived ease of use and user acceptance is greater than 0.50 and moderate. However, Mohamed et al [44] showed that the coefficient of determination must be larger than 0.19, the path coefficient between latent variables must be at least 0.1, and the significance level must be at least .05 in order to validate the model. Our CDSS evaluation model meets all these criteria, except the path coefficient from perceived ease of use to perceived user acceptance, which was 0.015. Hair et al [26], however, stated that path coefficients with standardized values greater than 0.20 are typically significant, while in this study, the path coefficient from perceived ease of use to user acceptance was 0.015, which is less than 0.10 and not significant. More information is depicted in Figure 9, which includes the details of the CDSS assessment PLS-SEM model developed from the CDSS Jimma Health Center and Jimma Higher Two Health Center evaluation data sets, including path weights, *P* values, and the coefficient of determination (R^2^).

Overall, as shown in Table 6, the PLS-SEM results suggested that the perceived ease of use has no positive effect on CDSS acceptance (β=.015, *P*=.91) but, rather, on system quality (β=.321, *P*=.002) and perceived benefits (β=.486, *P*<.001) of the CDSS. We also observed that information quality had a positive influence on the perceived ease of use (β=.678, *P*<.001). However, system quality had no favorable impact on the perceived ease of use (β=.021, *P*=.89). The detailed conclusions and summary based on PLS-SEM are shown in Table 6.

### Limitations

In this study, we evaluated our own proof-of-principle CDSS in LRSs. The small sample size and low number of cases in our study might limit the generalizability of our findings. As a result, difficulties that were not identified during this investigation may be identified during a longitudinal and case-by-case evaluation.

### Conclusion

We designed and developed a CDSS based on LRS requirements, which we evaluated in 2 LRSs in Ethiopia: the Jimma Health Center and the Jimma Higher Two Health Center. Our overall result indicates that user acceptance is impacted by system quality, information quality, perceived ease of use, and perceived benefits, with an R^2^ value of 0.703. Specifically, system quality and perceived benefits have a direct impact on user acceptance of the CDSS in LRSs. In this study, however, we found that the perceived ease of use and information quality had no positive effect on CDSS acceptability. Overall, the proposed acceptance model includes specific factors and variables, which is an important step toward the successful adoption and implementation of a CDSS in LRSs.

## Abbreviations

ANC: antenatal care
AVE: average variance extracted
CDSS: clinical decision support system
CG: clinical guideline
CP: clinical pathway
FDRE-MOH: Federal Democratic Republic of Ethiopia Ministry of Health
HTMT: heterotrait-monotrait ratio of correlations
LRS: low-resource setting
mHealth: mobile health
MS: measured symptom
PLS: partial least squares
PLS-SEM: partial least squares structural equation modeling
PNC: postnatal care
SEM: structural equation modeling
